# Electronic Health Informatics Data To Describe Clearance Dynamics of Hepatitis B Surface Antigen (HBsAg) and e Antigen (HBeAg) in Chronic Hepatitis B Virus Infection

**DOI:** 10.1128/mBio.00699-19

**Published:** 2019-06-25

**Authors:** Louise O. Downs, David A. Smith, Sheila F. Lumley, Meha Patel, Anna L. McNaughton, Jolynne Mokaya, M. Azim Ansari, Hizni Salih, Kinga A. Várnai, Oliver Freeman, Sarah Cripps, Jane Phillips, Jane Collier, Kerrie Woods, Keith Channon, Jim Davies, Eleanor Barnes, Katie Jeffery, Philippa C. Matthews

**Affiliations:** a Department of Infectious Diseases and Microbiology, John Radcliffe Hospital, Oxford University Hospitals NHS Foundation Trust, Oxford, United Kingdom; b Nuffield Department of Medicine, Medawar Building for Pathogen Research, University of Oxford, Oxford, United Kingdom; c NIHR Oxford Biomedical Research Centre, John Radcliffe Hospital, Oxford University Hospitals NHS Foundation Trust, National Institute for Health Research Health Informatics Collaborative, Oxford, United Kingdom; d Oxford NIHR Biomedical Research Centre Clinical Informatics Group, Big Data Institute, Li Ka Shing Centre for Health Information and Discovery, University of Oxford, Oxford, United Kingdom; e Pharmacy Department, John Radcliffe Hospital, Oxford University Hospitals NHS Foundation Trust, Oxford, United Kingdom; f Department of Hepatology, John Radcliffe Hospital, Oxford University Hospitals NHS Foundation Trust, Oxford, United Kingdom; Columbia University Medical College

**Keywords:** biomarker, health informatics, hepatitis B virus, surface antigen, viral clearance

## Abstract

HBsAg and HBeAg have gained traction as biomarkers of control and clearance during chronic hepatitis B virus infection (CHB). Improved understanding of the clearance correlates of these proteins could help inform improvements in patient-stratified care and advance insights into the underlying mechanisms of disease control, thus underpinning new cure strategies. We collected electronic clinical data via an electronic pipeline supported by the National Institute for Health Research Health Informatics Collaborative (NIHR HIC), adopting an unbiased approach to the generation of a robust longitudinal data set for adults testing HBsAg positive from a large UK teaching hospital over a 6-year period (2011 to 2016 inclusive). Of 553 individuals with CHB, longitudinal data were available for 319, representing >107,000 weeks of clinical follow-up. Among these 319 individuals, 13 (4%) cleared HBsAg completely. Among these 13, the HBsAg clearance rate in individuals on nucleos(t)ide analogue (NA) therapy (*n* = 4 [31%]; median clearance time,150 weeks) was similar to that in individuals not on NA therapy (*n* = 9 [69%]; median clearance time, 157 weeks). Those who cleared HBsAg were significantly older and less likely to be on NA therapy than nonclearers (*P* = 0.003 and *P* = 0.001, respectively). Chinese ethnicity was associated with HBeAg positivity (*P* = 0.025). HBeAg clearance occurred in individuals both on NA therapy (*n* = 24; median time, 49 weeks) and off NA therapy (*n* = 19; median time, 52 weeks). Improved insights into the dynamics of these biomarkers can underpin better prognostication and patient-stratified care. Our systematized approach to data collection paves the way for scaling up efforts to harness clinical data to address research questions and support improvements in clinical care.

## Abstract (precis)

Advances in the diagnosis, monitoring, and treatment of hepatitis B virus (HBV) infection are urgently required if we are to meet international targets for elimination by the year 2030. Here we demonstrate how routine clinical data can be harnessed through an unbiased electronic pipeline, showcasing the significant potential for amassing large clinical data sets that can help to inform advances in patient care and provide insights that may help to inform new cure strategies. Our cohort from a large UK hospital includes adults from diverse ethnic groups that have previously been underrepresented in the literature. By tracking two protein biomarkers that are used to monitor chronic HBV infection, we provide new insights into the timelines of HBV clearance, both on and off treatment. These results contribute to improvements in individualized clinical care and may provide important clues into the immune events that underpin disease control.

## INTRODUCTION

Chronic hepatitis B virus (CHB) infection is defined as detectable hepatitis B surface antigen (HBsAg) at ≥2 time points ≥6 months apart. Disease activity and treatment response in individuals with CHB infection are most commonly monitored by quantification of hepatitis B virus (HBV) DNA viral load (1). However, viral load measurement is expensive and not universally available, viral DNA levels can fluctuate over time, and quantification can be inaccurate at low levels. Reproducible, automated quantification of other biomarkers, such as HBsAg and/or hepatitis B e antigen (HBeAg), is therefore attractive for use instead of, or alongside, HBV DNA monitoring.

**FIG 1:**
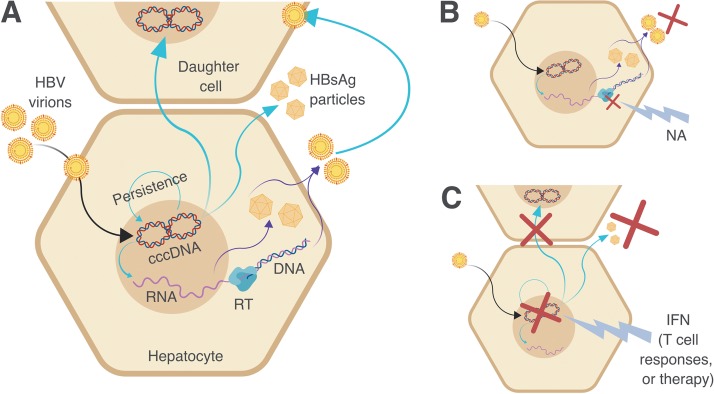
Cartoons depicting key pathways in the HBV replication cycle to illustrate targets that may bring about control or clearance. The figure was created using biorender.com. (A) Pathways relevant to the maintenance of HBV infection. HBV viral DNA is released in the nucleus, and cccDNA is formed by covalent ligation of the two DNA strands. A stable minichromosome is formed, allowing persistence of the virus over time. The cccDNA acts as the template for mRNA and pregenomic RNA (pgRNA). Viral reverse transcriptase (RT) generates new genomic DNA from pgRNA. Noninfectious subviral particles (SVP) form from HBsAg, and new infectious virions assemble, for release into the bloodstream. HBsAg measurement accounts for both the SVP and infectious virions, whereas infectious virions alone can be measured through HBV viral load (HBV DNA). (B) Pathways relevant to suppression of HBV infection by NA therapy. Inhibition of viral RT suppresses generation of new viral DNA. This means that new infectious HBV virions cannot be constructed and that HBV DNA is undetectable in plasma. However, cccDNA remains as a persistent reservoir in the hepatocyte nucleus, so HBsAg production can continue and rebound viremia is likely following cessation of therapy. For this reason, individuals with CHB on successful treatment frequently have an undetectable viral load but remain HBsAg positive. (C) Pathways relevant to functional or sterilizing cure of HBV infection. Upregulation of host immune responses or therapy with interferon (IFN) leads to elimination of the persistent cccDNA reservoir either through death of the hepatocyte or unknown nonlytic methods. HBsAg and HBV DNA both disappear from the bloodstream. In practice, there is no clinical test that can confirm complete (sterilizing) cure, so this group is usually regarded as being at a small risk of relapse (i.e., functional cure).

In the context of CHB infection, HBV covalently closed circular DNA (cccDNA) persists as an intranuclear “minichromosome” within infected hepatocytes (Fig. 1A). HBsAg is produced in excess, from translation from both the cccDNA reservoir and pregenomic mRNA (2). In a small proportion of cases, HBsAg becomes undetectable over time, suggesting that the cccDNA reservoir is diminished or suppressed. In the HBV field, specific terminology has been adopted to reflect the difference between complete loss of all cccDNA (“sterilizing cure”) and suppression or dilution of cccDNA to the extent that HBsAg can no longer be detected (“functional cure”) (3, 4). In practical terms, there is no current way to differentiate between these two outcomes. However, the theoretical distinction is an important one, as sterilizing cure reflects complete and permanent loss of HBV from the host, while in the setting of functional cure, there is long-term potential for relapse to occur (best recognized in the setting of immunosuppression) (5, 6).

Nucleos(t)ide analogues (NAs) inhibit HBV reverse transcriptase, leading to loss of HBV DNA from the serum, but have no direct effect on cccDNA (3). Thus, HBsAg production can continue unchecked from the cccDNA reservoir, and viral replication frequently returns upon cessation of treatment (Fig. 1B) (3). For this reason, most guidelines currently recommend long-term NA treatment (1). Immune responses (either arising naturally or driven by immunotherapy such as interferon [IFN] therapy) can lead to downregulation or loss of cccDNA to the extent that neither HBsAg nor HBV DNA can be detected in the serum (Fig. 1C) (4). The long-term goal of new immunotherapeutic approaches will be to elicit sterilizing cure such that cccDNA is removed with no long-term risk of relapse (7).

HBsAg levels are typically highest in the early phases of infection and in HBeAg-positive individuals, frequently correlate with HBV DNA levels in CHB infection, and are associated with a risk of subsequent reactivation (8). HBsAg may be a quantifiable risk factor for development of hepatocellular carcinoma (HCC) and chronic liver disease (9), although the relationship is not well defined: in some studies, higher HBsAg levels are associated with lower levels of fibrosis (10–12), while in others, lower baseline HBsAg levels are associated with reduced risk of both cirrhosis and HCC (13). HBsAg levels have also been used to classify individuals into those with inactive carriage (HBV DNA levels of <2,000 IU/ml and normal alanine aminotransferase [ALT] levels [14, 15]) versus those with active CHB (with higher viral loads and elevated risks of inflammatory liver disease, fibrosis, and cirrhosis [16–19]). HBsAg elimination is widely regarded as a marker of immunological clearance (which may be regarded as functional cure).

HBeAg positivity is associated with high viral loads and is therefore a marker of infectivity. Loss of HBeAg is usually associated with the production of anti-HBe antibody (a marker of immune-mediated control) and typically associated with lower viral loads. However, although these broad patterns have been described, further efforts are required to elucidate and interpret the dynamics of HBsAg and HBeAg, with the potential to develop insights into the timing and patterns of immunological clearance and to improve patient-stratified clinical management.

A recent systematic review/meta-analysis has collated literature on HBsAg clearance, with a primary focus on untreated populations (20). This review identified 34 studies, but only 14 of them reported two or more HBsAg measurements over time and all but two were in Asia. To ensure that we had adequately reviewed the relevant existing evidence on this topic, we also undertook an independent literature review (summarized in Table 1 at https://doi.org/10.6084/m9.figshare.7262957.v1). We initially identified 43 studies reporting the dynamics of HBsAg loss in CHB infection. We excluded studies prior to 2008, those reporting only one HBsAg measurement, and those without an annual or cumulative HBsAg clearance rate, leaving nine relevant studies. As for the meta-analysis, the majority of studies (8 of 9) were in Asian populations (21–28), with the remaining one based in New Zealand (29). The reported clearance rate of HBsAg ranged from 0.15% per year (27) to 2.7% per year (24), with a maximum cumulative clearance of 3.5% (21). Older age was associated with clearance in two cohorts (23, 29). The role of treatment in clearance is inconsistent, with NA treatment associated with clearance in some cohorts (21, 25) but not in others (26).

HBsAg levels can be used to determine treatment response, although this has been more reliably reported for pegylated IFN-2α (PEG-IFN-2α) treatment than for NAs (30, 31), as it implies reduction or removal of the cccDNA reservoir (Fig. 1C). Current UK guidelines recommend quantitative HBsAg and HBeAg measurements before starting treatment and at weeks 12, 24, and 48 during treatment, followed by 6 monthly measurements during long-term therapy (32). The European Association for the Study of the Liver (EASL) guidelines recommend quantitative HBsAg measurement annually in treated patients if HBV DNA is undetectable, as well as the use of HBsAg levels to inform the decision to stop treatment (1). EASL guidelines also recommend HBeAg measurement as part of the initial clinical assessment and list HBeAg loss as one of the serological responses to treatment but do not specify a frequency for follow-up testing (1).

International targets arising from the United Nations “sustainable development goals” have set a challenge for the elimination of CHB infection as a public health threat by the year 2030 (33). Recognizing the multilateral approaches that will be required to reach this ambitious goal, we here focus on two interrelated aims. (i) We set out to showcase how longitudinal data for individuals with CHB can be collected through an unbiased electronic pipeline that collates, cleans, and anonymizes routinely collected electronic clinical data, in this case driven by infrastructure supported by the UK National Institute for Health Research (NIHR) Health Informatics Collaborative (HIC) (https://hic.nihr.ac.uk). The aim is to harness clinical data to drive research and quality improvements in diagnostics, monitoring, and therapy of viral hepatitis and to underpin new questions for basic science. Through the development and testing of this system, we devised an approach that can be rolled out to incorporate other centers, with substantial gains predicted through the power of large data sets.

(ii) We analyzed data for HBV sourced from a tertiary referral UK teaching hospital in order to develop better insights into patterns of HBsAg and HBeAg clearance. Through the application of an unbiased approach (agnostic to treatment, clinical stage of disease, other biomarkers, or genotype of infection), we aim to develop a clear picture of the dynamics of clearance. Identifying demographic or clinical characteristics that predict specific disease outcomes provides opportunity for the investigation of immunological correlates of control and clearance.

Collectively, this enterprise provides proof of principle for the systematic use of electronic clinical data in informing studies of viral hepatitis, as well as shedding new light on the dynamics of clearance of HBsAg and HBeAg.

(An earlier version of this data set was presented as a poster at the EASL International Liver Conference, Paris, France, 2018 [34].)

## RESULTS

### Description of a clinical cohort of individuals with chronic HBV infection.

**FIG 2:**
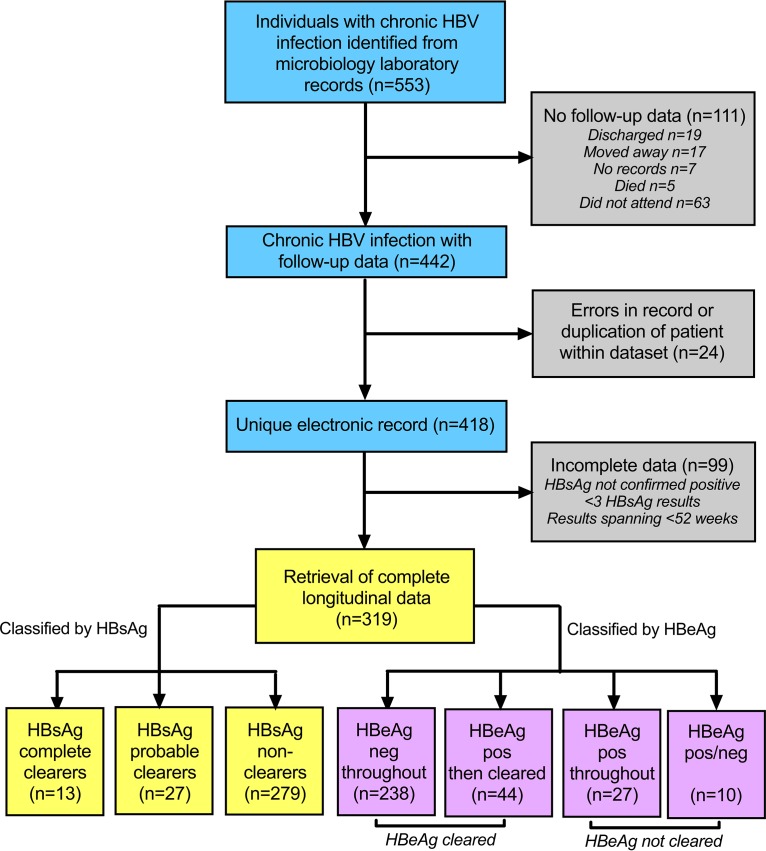
Flowchart showing identification and classification of adults with chronic HBV infection from a hospital electronic system. The figure represents 319 individuals who met inclusion criteria, which are divided into three different categories according to HBsAg clearance and four categories for HBeAg (for classification criteria, see Table 1).

We identified 553 individuals who tested HBsAg positive during the 6-year period from 2011 to 2016 inclusive. Of these, 319 met inclusion criteria for further analysis (Table 1 and Fig. 2). Characteristics of the cohort are summarized in Table 2, and the complete metadata for these 319 CHB patients is available as a supporting data file (see Table 2 at https://doi.org/10.6084/m9.figshare.7262957.v1). We collected longitudinal data for a total of 107,702 person-weeks (range, 61 to 702 weeks; mean, 338 weeks [6.5 years] of follow-up per individual; interquartile range [IQR], 174 to 487 weeks). The median age at first HBsAg test was 34 years (IQR, 29 to 43 years; range, 10 to 71 years), and males accounted for 191 of 319 (60%) cases. HIV coinfection was documented in 9 individuals (2.8%), although we cannot exclude the possibility that the true prevalence of HIV coinfection was higher due to a proportion of individuals who did not have a recent HIV test result.

**TABLE 1:** Summary of criteria used to confirm inclusion in the analysis and to classify individuals according to HBsAg and HBeAg dynamics

Category	Criteria
Inclusion in cohort for analysis	Unique electronic record available
	Age of ≥18 yrs at time of data interrogation
	Longitudinal laboratory data available
	No ambiguous data points^*a*^
	HBsAg detectable at ≥2 time points ≥6 months apart (HBsAg, >20 IU/ml)
	≥1 further HBsAg reading (either positive or negative) with a total surveillance period of ≥12 months
	
HBsAg categories	
HBsAg clearer	HBsAg initially detectable but subsequently falls below the limit of detection (<20 IU/ml)
	HBsAg does not rebound to ≥20 IU/ml
	≥2 consecutive HBsAg readings of <20 IU/ml
Potential HBsAg clearer	HBsAg falls to <1,000 IU/ml on ≥2 independent occasions
	HBsAg does not rebound to >1,000 IU/ml
	HBsAg not below the limit of detection for two consecutive readings
HBsAg nonclearer	All individuals who are not classified as HBsAg clearer or potential clearer
	
HBeAg categories	
HBeAg persistently positive	HBeAg above the limit of detection (≥20 IU/ml) for all time points
HBeAg persistently negative	HBeAg below the limit of detection (<20 IU/ml) for all time points
HBeAg clearer	HBeAg detectable at ≥2 independent time points and subsequently falls below the limit of detection for ≥2 consecutive time points
	HBeAg does not rebound above the limit of detection
HBeAg nonclearer	All individuals who are not classified as persistently HBeAg positive or negative or as an HBeAg clearer

a Records with free text or uninterpretable data were removed from analysis.

**TABLE 2:** Baseline characteristics of 319 individuals with CHB recruited through a UK cohort and classified according to pattern of HBsAg clearance over time^*a*^

Characteristic	Value for:				*P* value (univariable analysis)	*P* value (multivariable analysis)	Adjusted odds ratio	Adjusted 95% CI
	Whole cohort	HBsAg clearers and potential clearers		HBsAg nonclearers				
Total no. of individuals	319	40		279	NA	NA	NA	NA
Median age (yrs) at time of first HBsAg test	34	40		34	0.0034*	0.008*	0.96	0.93–0.99
Sex [no. (%) of individuals]								
Male (B)	191 (60)	26 (65)		165 (59)	B	B	B	B
Female	128 (40)	14 (35)		114 (41)	0.605	0.699	1.15	0.57–2.43
Self-reported ethnicity [no. (%) of individuals]								
White (B)	92 (29)	15 (38)		77 (28)	B	B	B	B
Mixed	18 (6)	0 (0)		18 (6)	0.986	0.986	UN	UN
Asian or Asian British	52 (16)	7 (18)		45 (16)	0.649	0.549	1.36	0.52–3.87
Black or Black British	46 (14)	5 (12)		41 (15)	0.396	0.383	1.63	0.57–5.39
Chinese	56 (18)	8 (20)		48 (17)	0.743	0.574	1.32	0.51–3.64
Any other ethnic group	7 (2)	0 (0)		7 (3)	0.991	0.991	UN	UN
Not stated	48 (15)	5 (12)		43 (15)	NA	NA	NA	NA
Other characteristics								
HBeAg-positive status at baseline [no. (%) of individuals]	81 (25)		6 (15)	65 (23)	0.3105	0.291	1.67	0.68–4.76
Median elastography score (kPa) (most recent value)	5.3		4.5	5.5^*b*^	0.18	NA	NA	NA
No. of individuals receiving treatment/total no. (%)	142/211^*c*^ (67)		11/40^*d*^ (28)	131/171^*c*^ (76)	<0.0001*	NA	NA	NA

a NA, not applicable. Elastography score and treatment were not included in multivariate analysis due to missing data for these variables. UN, numbers too low, so confidence intervals uninterpretable; B, base category in regression model. *, *P* value significant at <0.05.

b Elastography data were available for 42 individuals in the nonclearance group, as data were not routinely recorded electronically.

c Treatment data were missing for 108 individuals among the HBsAg nonclearers, as data were not routinely recorded electronically.

d Treatment in the 12 months before the last positive HBsAg test.

### Frequency of HBsAg clearance.

**FIG 3:**
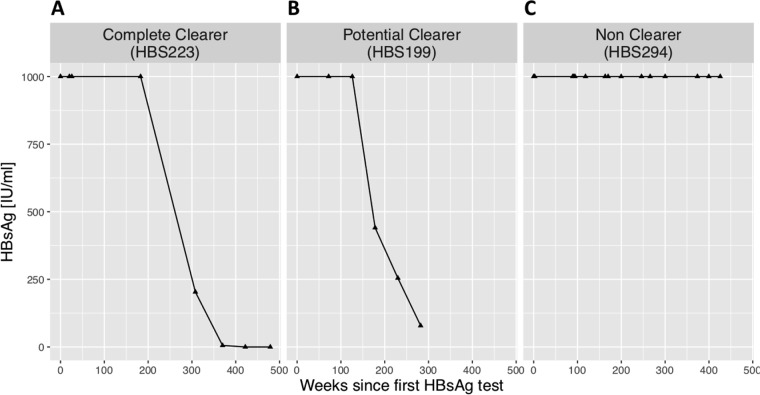
Examples of trajectories of HBsAg over time representing adults with chronic HBV infection. Individuals are classified as a complete HBsAg clearer (A), a potential HBsAg clearer (B), or an HBsAg nonclearer (C) (for classification criteria, see Table 1).

Examples of patterns of HBsAg clearance are illustrated in Fig. 3 (based on definitions given in Table 1). Using the most stringent definition of HBsAg clearance, we documented complete clearance in 13 of 319 (4.1%) individuals (for full details, see Table 2 and clearance trajectories in Fig. 1 at https://doi.org/10.6084/m9.figshare.7262957.v1). The HBsAg clearance rate for this cohort was 0.6% per year. In only 2 of 13 cases could we estimate the likely duration of infection prior to clearance, namely, in one individual who had been vertically infected (HBS-145) and in one with iatrogenic infection related to a blood transfusion in childhood (HBS-113). These individuals were both infected for approximately 25 years before clearing HBsAg.

We classified an additional 27 of 319 (8.5%) individuals as “potential clearers” on the grounds of HBsAg trends consistently declining toward clearance (criteria shown in Table 1; see Fig. 2 at https://doi.org/10.6084/m9.figshare.7262957.v1 for clearance curves). These potential clearers represent a more heterogenous group, but the clearance trajectory in all cases suggests that they would meet the more stringent clearance criteria if prospective surveillance were to be continued. In contrast are the HBsAg curves for nonclearers, which are shown in Fig. 3 at the URL shown above.

### Characteristics of individuals with HBsAg clearance or potential clearance.

**FIG 4:**
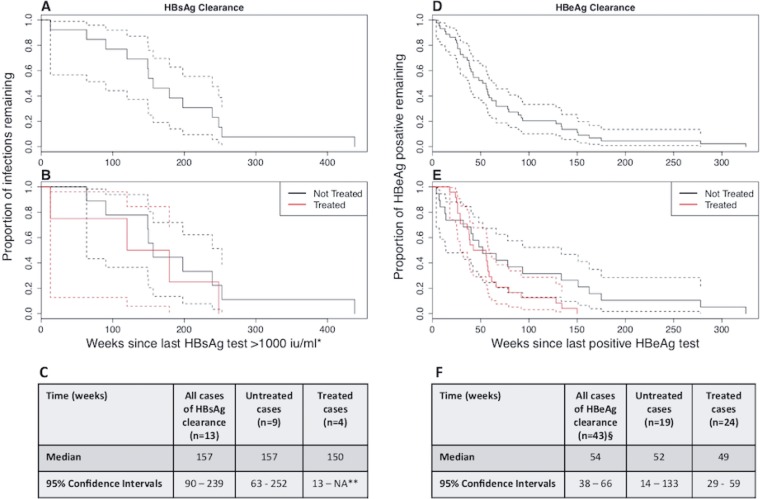
Kaplan-Meier curves showing trajectory of HBsAg clearance (*n* = 13) and HBeAg clearance (*n* = 43) for selected individuals who met criteria for complete clearance from within a cohort of adults with chronic HBV infection. Data are shown for HBsAg (A to C) and HBeAg (D to F), initially for all clearers (A and D) and then subdivided according to treatment status (B and E). Charts C and F report the median time to clearance for each group in weeks, with 95% confidence intervals. For HBsAg clearance, the upper confidence interval for treated cases could not be determined due to small numbers. Treatment of HBsAg clearers and potential clearers comprised tenofovir disoproxil fumarate (TDF) monotherapy (*n* = 3), TDF with emtricitabine (*n* = 2), lamivudine (3TC) with adefovir pivoxil (ADV) or TDF (*n* = 4), 3TC monotherapy (*n* = 1), and entecavir (ETV) monotherapy (*n* = 1). Treatment of HBeAg clearers comprised TDF monotherapy (*n* = 10), 3TC monotherapy (*n* = 2), ETV monotherapy (*n* = 5), 3TC with ADV (*n* = 3), interferon (IFN) with ribavirin (RBV) (*n* = 1), and IFN monotherapy (*n* = 3); treatment data were not available for one individual. *, when no values of >1,000 IU/ml were recorded, the highest value was used; **, not enough data to calculate the upper CI; §, treatment status not known for one individual.

Adults classified as completely or potentially clearing HBsAg were significantly older than nonclearers (median age, 40 versus 34 years, respectively; *P* = 0.003) (Table 2; see Fig. 4 at https://doi.org/10.6084/m9.figshare.7262957.v1). There was no difference in sex or ethnic origin between individuals in the different HBsAg clearance categories (Table 2). The majority of those who completely cleared HBsAg were HBeAg negative throughout the period of observation (10/13, 77%). Among the remaining three with detectable HBeAg, two of these lost HBeAg prior to clearing HBsAg (HBS-197 and HBS-223), while one (HBS-195) cleared HBsAg and HBeAg together (see Fig. 1 at https://doi.org/10.6084/m9.figshare.7262957.v1). In three cases (HBS-113, HBS-145, and HBS-195), HBV DNA was cleared at the same time as HBsAg; however, in the other 10 individuals (77% of clearers), HBV DNA levels were low (<100 IU/ml) throughout the period of HBsAg clearance.

### Rate of HBsAg clearance.

HBsAg clearance occurred over a median time of 157 weeks (95% confidence interval [CI], 90 to 239 weeks) (Fig. 4A). In a comparison of individuals on treatment (*n* = 4) with those off treatment (*n* = 9) during or in the 12 months prior to HBsAg clearance, clearance occurred over similar time frames (median of 150 weeks in those on treatment versus 157 weeks in those not on treatment) (Fig. 4B). Among 279 HBsAg nonclearers, 246 of 279 (88%) had HBsAg levels that were persistently >1,000 IU/ml. The remaining 12% had more heterogenous HBsAg dynamics, including transient dips to <1,000 IU/ml (e.g., HBS-298) and sustained levels of <1,000 IU/ml but without a trend toward clearance (e.g., HBS-368).

### Treatment status of HBsAg clearers versus nonclearers.

During the HBsAg clearance phase, or in the 12 months prior, 4 of 13 (31%) individuals defined as having completely cleared HBsAg were on NA therapy (Fig. 4B and C). These individuals had received treatment for a median of 13 months (range, 2 months to 8 years) prior to clearance. The other nine (69%) had not received treatment in the 12 months prior to HBsAg clearance, but one had received PEG-IFN-2α therapy 4 years earlier. In those individuals defined as potential clearers, 7 of 27 (26%) received NA treatment, two of whom received tenofovir disoproxil fumarate (TDF) as part of an HIV treatment regimen.

We also reviewed treatment data for the 279 individuals who did not clear HBsAg and were able to retrieve data for 171 (61%) of these. Among these, 131 (77%) had received treatment of some type, and 40 had never been treated (23%). We were not able to determine robust time frames for most treatment episodes. Based on these data, HBsAg nonclearers were statistically more likely to be on treatment than HBsAg clearers (131 of 171 versus 4 of 13, respectively, *P* = 0.001 by Fisher’s exact test). This may reflect inherently better immune control in the group who clear HBsAg, meaning that they are less likely to meet criteria for treatment than nonclearers. However, these data must be interpreted with caution, as bias is introduced as a result of missing data among the nonclearers and by different timelines for follow-up (we assessed treatment cross-sectionally in clearers based on a specific time of HBsAg loss, for which there is no equivalent among nonclearers, and thus we may have assessed longer follow-up times in the latter group).

### HBeAg status.

HBeAg was detectable in 81 of 319 (25%) individuals at the start of the observed time period. Among these, 51 of 81 (63%) were male and the median age was 34 years. By univariable regression analysis, Chinese ethnicity was associated with HBeAg-positive status, with 22 of 56 (39%) Chinese individuals being HBeAg positive (*P* = 0.049; odds ratio [OR] = 2.05; CI, 1.003 to 4.25; in a comparison with the largest other ethnicity group [white] as the base category). We documented HBeAg clearance in 44 of 81 (54%) of these individuals over the observed time period (Table 3) at a median age of 37 years. HBeAg loss occurred over a median period of 54 weeks (95% CI, 38 to 66 weeks) between the last positive and the first negative HBeAg test (Fig. 4D). Median clearance was 49 weeks (95% CI, 29 to 59 weeks) for individuals who had received treatment in the year prior to the last positive HBeAg result (*n* = 24, 55%) and 52 weeks (95% CI, 14 to 133 weeks) for untreated individuals (*n* = 19, 43%); treatment data were not available for one individual (Fig. 4E and F). We also reviewed treatment data for those who did not clear HBeAg and were able to retrieve data for 27 of these (73%). Of these, 24 (89%) had received some treatment, while 3 (11%) were untreated.

**TABLE 3:** Baseline characteristics of HBeAg-positive individuals classified according to HBeAg clearance over the observed time period^*a*^

Characteristic	Value for:		*P* value (univariable analysis)
	HBeAg clearers	HBeAg nonclearers	
Total no. of individuals	44	37	
Median age (yrs) at time of first HBsAg test	34	35	0.75
Sex [no. (%) of individuals]			1
Male	29 (66)	25 (58)	
Female	15 (34)	12 (32)	
Self-reported ethnicity [no. (%) of individuals]			
White	12 (27)	10 (27)	B
Mixed	4 (9)	4 (11)	0.680
Asian or Asian British	8 (18)	3 (8)	0.996
Black or Black British	6 (14)	0 (0)	0.997
Chinese	8 (18)	14 (38)	0.822
Any other ethnic group	1 (2)	2 (5)	0.999
Not stated	5 (11)	4 (11)	NA
Median elastography score (kPa) (based on most recent value)	5.5	4.55	0.24
No. of individuals receiving treatment/total no. (%)^*b*^	24/44 (55)	24/27^*c*^ (89)	NA

a NA, not applicable; B, base category in regression model.

b Treatment in the 12 months prior to the last positive HBeAg result.

c Treatment data were missing for 10 individuals among the HBeAg nonclearers, as data were not routinely collected electronically.

### Association between HBsAg clearance and ALT.

Complete longitudinal ALT data are shown for each individual in Fig. 1 to 3 at https://doi.org/10.6084/m9.figshare.7262957.v1. We investigated whether there were differences in ALT levels according to HBsAg clearance (for each of the three HBsAg groups defined in Fig. 2). There was no significant difference in ALT level at the time of first test between HBsAg clearers, potential clearers, and nonclearers (data not shown). ALT data were available before and during HBsAg clearance for 11 of 13 individuals who cleared HBsAg. Among these, three individuals (HBS-162, HBS-195, and HBS-314) had a spike in ALT before clearance that returned to the normal range after HBsAg clearance. Another individual (HBS-230) also had a slightly raised ALT level before HBsAg clearance, but this did not normalize after HBsAg clearance. In the 7 other cases, ALT results remained within the local reference range (10 to 45 IU/liter) for the entire period of surveillance (see Fig. 1 at https://doi.org/10.6084/m9.figshare.7262957.v1).

### Relationship between HBsAg and HBV DNA.

In 11 of 13 HBsAg clearers, HBV DNA was below the limit of detection (<20 IU/ml) throughout; in two cases, HBV DNA was cleared at the same time as HBsAg (see Fig. 1 at the URL shown above). The HBV DNA trajectory of individuals classified as potential clearers was more heterogenous (see Fig. 2 at the URL shown above): 10 individuals had cleared HBV DNA by the time of their last HBsAg test, 9 had negative HBV DNA results at some point but had subsequent detectable viremia, and 8 individuals had detectable HBV DNA throughout the period of surveillance.

## DISCUSSION

### Novelty.

HBsAg clearance in CHB infection is an uncommon event, and large cohorts over a long period of clinical follow-up are therefore required to describe the characteristics of individuals who clear and to determine the specific dynamics of serological changes. Although there have been previous studies reporting HBsAg loss, our literature review confirms that these are mostly focused in Asia and that relatively few studies track longitudinal data in an unbiased way. Our current approach adds novelty in a variety of ways, as follows. (i) We apply a new bespoke, algorithmic approach to the collation of a large longitudinal clinical data set from multiple electronic sources. This allowed us to make use of data that are generated by routine clinical laboratories but are not routinely used for patient care, such as quantitative measures of HBsAg and HBeAg. This method of data collection also facilitated the robust identification and exclusion of duplicate patient records.

(ii) Undertaking this analysis in a UK-based cohort provides a novel and more diverse mixture of host ethnicities (and by inference, diverse viral genotypes). To our knowledge, this is one of the only studies of this kind in such a population.

(iii) We report ≥2 HBsAg time points for each individual, providing long periods of clinical follow-up and the opportunity to track uncommon clearance events over time.

(iv) Unlike some previous studies of HBsAg clearance that introduce bias through a focus on treatment or based on patient recall for follow-up, the approach we took is agnostic to other parameters, thereby providing a more inclusive picture of all individuals with CHB infection.

(v) In addition to reporting longitudinal data for HBsAg loss, we also track HBeAg loss over time. HBeAg loss is an important immunological event (35) signifying control (typically in association with a decrease in HBV DNA levels) and may also be an important target for interventions at a population level (36).

### The value of the NIHR HIC approach.

The NIHR HIC approach, involving the generation of standardized data sets based upon routinely collected data that are focused on the needs of researchers in particular clinical and/or therapeutic areas, supports the reuse of tools, data, and insights across multiple research projects and organizations. As we address a wider range of questions, and as we continue to share expertise with other university-hospital partnerships, we will increase the breadth, depth, and quality of the data set, covering a wider range of variables, for a larger patient population, and recording more information about the provenance and interpretation of the values obtained. All of the data used for this paper, and our understanding of those data, will be available for use by other researchers. Any questions that we were unable to fully address, and other questions that emerged during the analysis, will help to inform the future development of the data set.

### Role of treatment in HBsAg clearance.

Our data set corroborates earlier literature in confirming that treatment is not prerequisite for clearance and that immunological clearance of HBsAg and HBeAg can occur independently of antiviral therapy (Fig. 1C) (23, 37). There are multiple host and viral factors influencing outcome during CHB infection, including host factors such as age, obesity, gender, and diabetes, along with genetic variations in CD8^+^ T-cell responses (mediated by HLA genotype), T-cell receptor antagonism, and viral escape mutations (38, 39).

Due to the small numbers of individuals, we did not have the statistical power to determine whether there was a significant difference in time taken to clear either HBsAg or HBeAg in individuals on treatment compared to those in an untreated group. However, the comparable speed of clearance on treatment and off treatment suggests that clearance trajectories are similar irrespective of NA treatment. We found that NA treatment was more common among nonclearers, which may reflect a genuinely higher proportion of this group meeting treatment criteria but may also be biased by the incomplete nature of our treatment data. Further prospective studies are needed to study the relationship between clearance and treatment in more detail.

### Timing of HBsAg and HBeAg clearance.

Based on the epidemiology of HBV infection in this cohort, in which a substantial proportion of individuals are likely to have been infected at birth or in early childhood, it is intriguing that HBsAg and HBeAg clearance occur apparently at random in middle adulthood. In the case of HBsAg clearance, its association with older age has been previously reported in studies we identified through our literature review (20, 23, 29). The chances of clearance may be cumulative over time; individuals infected for longer periods of time might thus have a higher chance of clearance, which could explain why individuals who clear infection are, on average, older than nonclearers.

HBeAg clearance occurred over a median period of 54 weeks, substantially more quickly than HBsAg clearance, which was documented over a median period of 157 weeks, perhaps indicating different underlying mechanisms at play (35, 38, 40). Further studies are needed to determine the relevant immune responses that underpin this clearance and to identify possible triggers for clearance.

### Relevance of HBsAg and HBeAg for clinical practice and research.

While some guidelines recommend monitoring of HBsAg levels (1, 32), there is a lack of consistent understanding about how to interpret individual or longitudinal measurements. Developing better insights into the prognostic information that can be captured from this biomarker could be relevant to predicting patient outcomes and providing stratification of therapy. In this study, we did not have routine access to HBsAg levels of >1,000 IU/ml, but as these data progressively become available, future studies will have the opportunity to develop a better picture of HBsAg distribution across the whole range of CHB infections. Advocacy is required to provide more universal access to platforms that quantify HBsAg and to improve clinical practice through interval measurements of HBsAg in chronically infected patients.

The picture we have developed here suggests that the majority of individuals who develop a sustained pattern of HBsAg decline below 1,000 IU/ml are likely to go on to clear HBsAg, consistent with previous longitudinal surveillance suggesting that baseline HBsAg levels may be a more accurate prognostic marker than HBV viral load (28, 29). Prospective studies of large HBV cohorts are likely to be needed to identify individuals on a clearance trajectory; enhanced surveillance of these individuals is a promising future route to understanding the immunological correlates of HBsAg clearance.

### Caveats and limitations.

Routinely collected clinical data may be lacking in context, consistency, and completeness. Health professionals recording information to support decision-making and continuity of care, and the systems that they use, may fail to record additional, contextual information needed to address specific research questions. Variations in practice may mean that data from different sites, or from different clinicians, are incompatible (Table 4). A collaborative approach to data quality improvement, with substantial, local clinical engagement, will help to address these challenges, but there is always more to be done as practice changes and new research questions evolve. For this paper, the questions that we were able to ask and the size of the population considered were limited by the nature and means of the data recorded, rather than by the basic availability. We considered only those clinical records for which the data were sufficiently complete and for which the context was adequately explained, accepting the possibility that our exclusion of other records could introduce systematic bias.

**TABLE 4:** Factors influencing the analysis of retrospective clinical HBV data

Category of influence	Examples of effect on data integrity
Patient factors	Many individuals with CHB infection globally are not diagnosed; those with data available for clinical analysis represent a distinct minority group who have been able to access healthcare and follow-up (44).
	Patients are lost to follow-up or move between regions.
	HBV diagnosis rarely occurs in acute infection, so the duration of infection prior to clearance is unknown.
	HBsAg clearance is a relatively infrequent event, and thus patient numbers for analysis are small.
	Description of a changing cohort is challenging (e.g., age changes over time, patients start and stop therapy).
Healthcare factors	Different assays are not always requested simultaneously, thus limiting the correlation between variables (e.g., HBV DNA versus HBsAg).
	Follow-up occurs over a variety of different time frames, with different intervals between follow-up visits; clearance durations may therefore be overestimated due to infrequent sampling.
	Treatment can alter the dynamics of biomarkers (e.g., ALT, HBV DNA).
Laboratory factors	Assay platforms change over time, which may alter sensitivity, specificity, and limits of detection.
	Quantitative assays have upper and lower limits of quantification; values outside the window of detection cannot be analyzed.
	False-positive or false-negative tests may occur.
	Certain data are not routinely generated or captured (e.g., HBV genotype).
Data factors	Results are captured by a variety of different electronic systems (e.g., electronic patient record, electronic laboratory systems, pharmacy systems, hand-written clinical notes, or dictated clinic letters).
	Different healthcare professionals may not record data consistently, and coding is subject to errors.
	Free-text entries in laboratory reporting can lead to errors or ambiguities (e.g., use of a comma versus a period for a decimal point).
	Certain parameters are not consistently recorded (e.g., ethnicity).
	The electronic pipeline collects only certain predefined data (e.g., for HIV, hepatitis C virus, and hepatitis D virus, we were able to access only viral load data, not antibody tests, and therefore we do not know the denominator of total tests performed).
	Treatment data may not be recorded electronically (often recorded as part of paper notes, making them more difficult to trace); start dates are often not documented for patients undergoing long-term treatment.
	Poor continuity of data when patients are transferred between different healthcare providers.

### Future questions.

Prospective surveillance is important in order to provide the opportunity for studying relevant immune responses during HBeAg and HBsAg clearance. As we have shown that clearance is a relatively long process, occurring over a median of 54 weeks for HBeAg and 157 weeks for HBsAg, this provides a window of opportunity for sampling and follow-up. There is an important distinction to be made between functional cure (sustained loss of HBsAg) and sterilizing cure (loss of cccDNA within hepatocyte nuclei) as well as interest in how to determine these different outcomes. There are currently many new therapeutics in clinical trials aimed at targeting cccDNA directly, including capsid effectors, RNA interference, and gene editing (7). Further work is needed to develop biomarkers that can detect cccDNA in order to distinguish between these two different outcomes.

Studies of both host and viral genetics are required to underpin a better understanding of the mechanisms of clearance, including new approaches to generating full-length deep sequencing of HBV and unbiased methods to study host genetic polymorphisms that impact disease outcome. In order to power such studies sufficiently to detect relevant signals, large collaborative multicenter studies may be required. As we improve our insights into the dynamic changes of serological markers, opportunities arise for improving prognostication and providing better patient-stratified care.

## MATERIALS AND METHODS

### HIC infrastructure.

The UK National Institute for Health Research (NIHR) Health Informatics Collaborative (HIC) (https://hic.nihr.ac.uk) is a program of infrastructure development aimed at increasing the quality and availability of routinely collected clinical data for translational research. Eighteen university-hospital partnerships across England have signed a framework data sharing agreement, and are working to facilitate the sharing and reuse of data across centers, for approved research purposes. A key component of the NIHR HIC approach is the creation of standardized data sets to support research in specific therapeutic areas, with relevant variations in context and practice recorded as structured metadata, facilitating reuse at scale. Viral hepatitis was selected as one of the initial areas for infrastructure development: in Oxford, this has led to the establishment of new data flows from clinical and laboratory systems, the design of new screens for data capture, and the implementation of several, programmatic (and reusable) data transformations.

### Clinical cohorts.

Our HBV cohort was collected from the records of a large UK teaching hospital in Oxford (http://www.ouh.nhs.uk/), which provides 1 million patient contacts per year and receives laboratory samples from the community and four inpatient sites. We retrospectively identified individuals aged ≥18 years at the time of database interrogation (26 March 2018) with chronic HBV infection (defined as HBsAg positivity on ≥2 occasions ≥6 months apart) based on laboratory data collected between January 2011 and December 2016. Inclusion criteria and other case definitions are set out in Table 1. It is standard practice in Oxfordshire to start patients on HBV treatment based on UK NICE guidelines, which determine treatment eligibility using age, sex, ALT, HBV DNA, and FibroScan score (32).

### Data collection.

**FIG 5:**
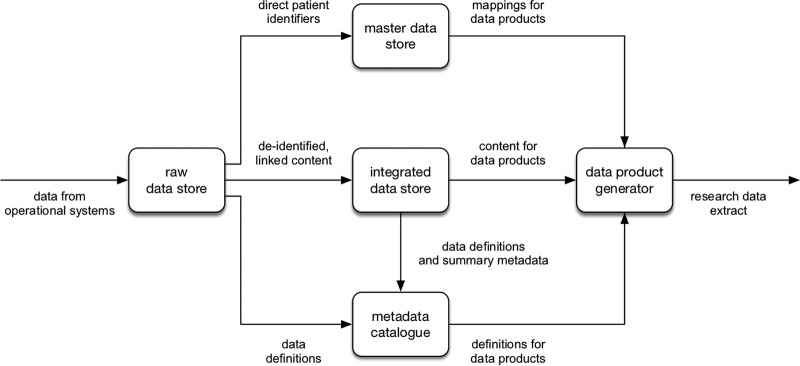
Flow diagram to depict collection, storage, and output of electronic clinical data from a Health Informatics Collaborative data warehouse. The data warehouse receives data from operational systems within the hospital, such as electronic patient records and laboratory information management systems (LIMS), and maps these data to individuals, whose identifiers are then stored in the master data store and provide the mappings for data products. Deidentified linked data are stored separately and form the content of data products. Definitions of data items are recorded in the metadata catalogue. Data items for the data product are selected using the definitions in the metadata catalogue, the mappings for these are retrieved from the master data store, and data are retrieved from the integrated data store to create the final data product.

Our cohort was initially defined by an electronic search of the microbiology laboratory information management systems (LIMS) to identify individuals with a positive HBsAg test. Individual subjects were allocated a pseudoanonymized identification (ID) number prefixed with “HBS,” and these ID numbers are included in the text to allow relevant results to be identified from within our metadata table (see Table 2 at https://doi.org/10.6084/m9.figshare.7262957.v1). We generated a data specification using search terms (Table 5) to define the data set. The Oxford NIHR Biomedical Research Centre (BRC) data warehouse receives data from operational systems within the hospital, such as electronic patient records (EPR) and LIMS (Fig. 5). Within the warehouse, the data are linked, transformed, and reorganized to better support the generation of data products focused on a particular purpose or research area. In this case, the data product is a database containing deidentified information on patients with hepatitis. These data were cleaned, and individuals not meeting inclusion criteria (Table 1) were removed.

**TABLE 5:** Data dictionary of clinical and demographic parameters collected for cohort of individuals with chronic HBV infection^*a*^

Laboratory parameter	Data source	Date range (mo/yr) for laboratory parameter	Assay platform and date range (mo/yr)	Notes
HBsAg	Microbiology LIMS (Sunquest)	09/2004– 03/2018	Centaur (09/2004–12/2014); Abbott Architect i2000SR (Abbott Laboratories, Chicago, IL) (12/2014–03/2018)	Traditionally reported as a binary test (positive/negative) but generates semiquantitative data; lower limit of detection, 0.05 IU/ml
HBeAg	Microbiology LIMS (Sunquest)	04/1995– 03/2018	Centaur (09/2004–12/2014); Abbott Architect i2000SR (Abbott Laboratories, Chicago, IL) (12/2014 – 03/2018)	Traditionally reported as a binary test (positive/negative) but generates semiquantitative data
HBV DNA	Microbiology LIMS (Sunquest)	03/2009– 03/2018	Cobas TaqMan assay (Roche Diagnostics, Branchburg, NJ)	Lower limit of detection, 0.9 × 10^1^ IU/ml; 1 IU/ml is equivalent to 2.5 to 5 genome equivalents (copies/ml)
ALT	Biochemistry LIMS (LIMS)	02/2013– 01/2018	Siemens ADVIA 2400 (02/2013–01/2015); Abbott Architect c16000 or c8000 (Abbott Laboratories, Chicago, IL) (01/2015–01/2018)	Reported as a quantitative value; normal reference range, 10 to 45 IU/liter^*b*^
Ethnicity	Hospital EPR (Cerner Millennium)	NA	NA	Self-reported according to standardized ethnicity codes
FibroScan result (transient elastography score)	Hospital EPR (Cerner Millennium)/clinic letter database (manual)	NA	Echosens, Paris, France	Most recent recorded elastography result
HBV treatment status	Hospital EPR (Cerner Millennium)/clinic letter database (manual)	NA	NA	Treatment guidelines changed over time, so use of different agents was applied across the timespan of the cohort

a LIMS, laboratory information management system; EPR, electronic patient record; NA, not applicable.

b In our hospital, no distinction is made in the ALT reference range for males versus females.

We devised classification criteria for HBsAg and HBeAg to sort each individual into a category based on the dynamics of these serologic markers (Table 1). For HBsAg and HBeAg clearers and HBsAg potential clearers, data which were not captured electronically or were not available from the data warehouse (e.g., most recent transient elastography score and HBV treatment status) were retrieved from the patient’s written clinical record or from dictated letters from the viral hepatitis clinic.

### Ethics.

The NIHR HIC Viral Hepatitis database was approved by the NRES Committee South Central-Oxford C on 6 October 2015 (REC reference no. 15/SC/0523).

### Statistical analysis.

We cleaned and analyzed data using R and the data.table package (41). The clearance rate was calculated as [(number of patients who cleared)/(total patient years)] × 100. Plots were created using ggplot2 (42), and survival analysis and Kaplan-Meier plots were created using the survival and rms packages (43). We used Wilcoxon or Kruskal-Wallis tests for mean comparison of continuous variables, Fisher’s exact test for comparison of categorical variables, and logistic regression for multivariable analysis. We included all the parameters in our data set in multivariable analysis, based on existing biological reasons to believe them likely to be relevant. Specifically, age at first HBsAg test is known to be associated with HBsAg clearance, sex and ethnicity could indicate differences in host genetics and immune response, and HBeAg status is a known marker of disease severity (20, 26, 38). The code used for this analysis is available in the HBsAg_Final_Analysis.html file at https://doi.org/10.6084/m9.figshare.7262957.v1. To define HBsAg clearance time frames, we measured from the time of the last HBsAg result of >1,000 IU/ml (or the result closest to 1,000 IU/ml) to the time point at which HBsAg first became undetectable. For analysis of ALT, we used the result corresponding to the time of the first HBsAg test result.

### Data availability.

Anonymized clinical metadata, source code used for analysis, and supplemental figures and tables are available at figshare (https://doi.org/10.6084/m9.figshare.7262957.v1).
